# Diffusion-weighted imaging in pediatric extracranial germ cell tumors

**DOI:** 10.1371/journal.pone.0294976

**Published:** 2023-11-30

**Authors:** Carlos Eduardo Bezerra Cavalcante, Fernanda Magalhães Pereira Souza, Gisele Eiras Martins, Marcelo Milone Silva, Carla Renata Pacheco Donato Macedo, Henrique Lederman, Luiz Fernando Lopes

**Affiliations:** 1 Department of Pediatric Radiology, Children’s Cancer Hospital, Hospital de Amor, Barretos, São Paulo, Brazil; 2 Pediatric Oncology, Children’s Cancer Hospital, Hospital de Amor, Barretos, São Paulo, Brazil; 3 Brazilian Germ Cell Pediatric Study Group, Hospital de Amor, Barretos, São Paulo, Brazil; 4 Centro de Tratamento Fabiana Macedo de Morais/GACC, São Jose dos Campos, São Paulo, Brazil; 5 Instituto de Oncologia Pediatrica - GRAACC, Universidade Federal de São Paulo, São Paulo, Brazil; 6 Department of Radiology, Grupo de Apoio ao Adolescente e à Criança com Câncer (GRAACC), Sao Paulo, Brazil; 7 Chairman, Brazilian Germ Cell Pediatric Study Group, Hospital de Amor, Barretos, São Paulo, Brazil; University of Bremen: Universitat Bremen, GERMANY

## Abstract

**Background:**

Germ cell tumors (GCTs) comprise a rare and heterogeneous group of neoplasms presenting different clinical and histological characteristics, leading to a challenging scenario in clinical practice. Diffusion-weighted imaging (DWI) has been suggested as an indirect marker of tumor density and cellularity and could be used to monitor therapeutic response. However, its role in pediatric GCTs needs to be clarified.

**Purpose:**

Here, we evaluated the features of DWI in pediatric extracranial GCTs in a reference Brazilian institution.

**Material and methods:**

We included 43 pediatric patients with primary GCTs treated between 2008 and 2022 in Hospital de Amor de Barretos. The patients’ MRI images included T1-weighted without contrast, T2-weighted, DWI and apparent diffusion coefficient (ADC) maps. DWI was evaluated in the section that exhibited the greatest restricted diffusion in the largest hypersignal area of the image. The lowest ADC value was determined to define the region of interest (ROI). We used a small ROI, avoiding necrotic, adipose tissue, noisy or nonenhancing lesion voxels as recommended. ROI determination was established by visual inspection by two radiologists in accordance. We used two values of b (b = 50 mm^2^/s or b = 800) for ADC values.

**Results:**

The highest mean ADC (mADC) value was observed in pure teratomas (1,403.50 ± 161.76 x10^-3^ mm^2^/s; mean ± SD) compared to other histologies (yolk sac, mixed teratoma, dysgerminoma and mixed GCT) of GCT (p<0.001). Furthermore, ROC analysis determined a cutoff mADC value of 1,179.00 x 10^−3^ mm^2^/s that differentiated pure teratomas from the other GCT histologies with a sensitivity of 95.8% and a specificity of 92.9% (AUC = 0.979; p<0.01). A significant increase in mADC was observed for malignant GCTs in treatment (1,197.00 ± 372.00 mm^2^/s; p<0.001) compared to that exhibited at the time of diagnosis (780.00 ± 168.00 mm^2^/s; mean ± SD. Our findings suggest that mADC assessment could be used as a tool to distinguish pure teratomas from malignant CGT histologies at diagnosis. Additionally, we demonstrated reasonable evidence that it could be used as a complementary tool to monitor treatment response in patients with malignant GCT.

## Introduction

Germ cell tumors (GCTs) comprise a rare and heterogeneous group of benign or malignant neoplasms that are clinically and pathologically complex, derived from primordial germ cells and can occur in gonadal or extragonadal sites [1]. GCTs present two distinct peaks of incidence, one before two years old, reflecting the high incidence of tumors with sacrococcygeal location, and the other between 8 to 12 years for females and 11 to 14 for males, representing the high incidence in ovarian and testicular tumors for this age group [1, 2]. GCTs represent 3.3% of malignant tumors in children and adolescents (<15 years). The annual occurrence is 0.4 cases per 100,000 children under 15 years old for malignant tumors and 0.6 cases per 100,000 children for teratomas [3–6]. Among children under five years old, teratomas are the most frequent histological subtype, followed by yolk sac tumors, germinomas and others. Regarding anatomical distribution, GCTs in childhood more often affect the sacrococcygeal region, followed by the gonadal region and others [1, 7, 8].

Treatment recommendations for pediatric germ cell tumors are difficult due to the inability to define widely accepted risk groups. The Brazilian Germ Cell Pediatric Study Group (BGC-PED Study Group) has shown over the years the importance of establishing a standard treatment protocol for improving patient survival, reaching a global 5-year survival rate greater than 80% for high-risk patients treated with cisplatin and etoposide [2]. Although the majority of GCTs present a good response to treatment with an effective cure potential as demonstrated, cases refractory to treatment lead to a challenging scenario in clinical practice [9].

A central question in the management of tumors concerns the ability to measure the response to treatment, leading to the optimization of therapy, mainly for those presenting resistant tumors [10, 11]. Currently, strategies for accessing treatment response are based on the identification of anatomical and morphological changes occurring in tumor size [12] as well as tumor serum markers [13].

In this context, diffusion-weighted imaging (DWI) has been suggested as an indirect marker of tumor density and cellularity and has been associated with injury aggressiveness and tumor response [14]. Even during successful anticancer therapy, changes in cell density due to necrosis and apoptosis cause significant changes in water diffusion, which can be detected by DWI. In addition, these changes occur early in relation to macroscopic response indicators, such as tumor size and volume [11, 15]. Quantitative analysis is also possible by calculating the apparent diffusion coefficient (ADC), assigning absolute values to the region under study. The increase in ADC values during treatment may indicate cell death [16], and both have been proposed as biomarkers of therapeutic response in several types of tumors, including brain, kidney, breast, liver, musculoskeletal, head and neck, prostate and uterine tumors [17, 18].

Potentially, DWI allows the identification of small changes in cell integrity at an early stage of treatment, which can be used to evaluate patients with GCT [19]. Additionally, DWI-derived parameters are attractive as image biomarkers because acquisition is noninvasive, does not require the use of contrast agent, does not use ionizing radiation, is quantitative and is obtained relatively quickly and easily incorporated into the patient’s routine [14], mainly in children. However, its role in managing pediatric extracranial GCTs is still poorly explored.

For this purpose, in this study, we evaluated DWI and ADC in MRI images available for 43 pediatric patients with primary extracranial GCTs treated according to the BGC-PED Study Group protocols in a single Brazilian pediatric cancer institution.

## Material and methods

The study was conducted following national and institutional ethical policies and was approved by the Hospital de Amor Ethical Committee (protocol CAAE 78263417.7.1001.5437). The ethics committee considered this retrospective study to have minimal risk, ensuring confidentiality and not resulting in any clinical implications for the patients. For these reasons, the ethical committee waived the need for informed consent.

### Patients and imaging protocol

We included 43 pediatric patients (age ≤ 21 years old; both sexes) diagnosed with primary germ cell tumors (gonadal or extracranial) treated at Children’s Cancer Hospital—Hospital de Amor, Barretos. All patients were enrolled in the treatment protocols of the BGC-PED Study Group, GCT-2008 or GCT-2017.

According to BGC-PED Study Group protocols, for patients with suspected GCTs, initial diagnostic investigation includes radiological images for the evaluation of staging, response to chemotherapy and the possibility of a surgical approach. Subsequently, the specific location of the tumor and the potential for dissemination are considered for a diagnostic evaluation. Clinical and radiological evaluation unequivocally indicating a circumscribed and localized tumor indicates complete surgical resection as the treatment of choice, minimizing risks for the patient. If metastatic disease and/or risk of mutilating surgery is confirmed, biopsy for histopathological diagnosis should be performed. The diagnosis of GCT is confirmed through microscopic examination of HE (hematoxylin and eosin) and immunohistochemical examination after obtaining tumor material by complete surgical resection of the tumor or biopsy. The GCT-2008 or GCT-2017 protocols use FIGO (International Federation of Gynecology and Obstetrics) staging for ovarian tumors [20] and COG (Children’s Oncology Group) staging for testicular and extragonadal tumors [21]. Data regarding tumor histology, primary site, surgery/biopsy and tumor size are demonstrated in S1 Table.

We evaluated MRI scans with DWI and ADC maps of the primary tumor at diagnosis available for patients in the period between 2008 and 2022. We included patients naive to treatment and patients who started treatment and were undergoing a reevaluation during treatment. Regarding teratomas with malignant components, we considered the histological subtypes of GCT as only germinoma, yolk sac tumor, embryonic carcinoma and choriocarcinoma.

### Image analysis

**Fig 1:**
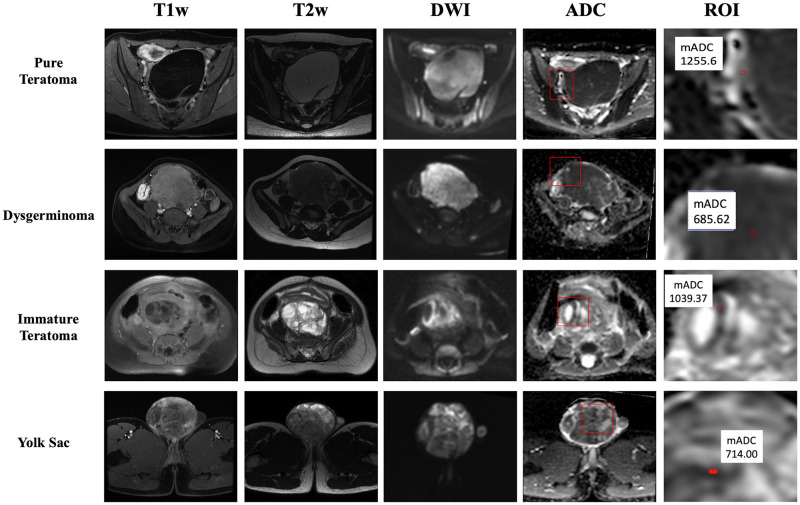
T1w, T2w, DWI and ADC maps of 4 representative patients with germ cell tumors. Hypersignal areas are observed in the diffusion sequence (DWI) showing correlation with areas of signal loss on the ADC map (ADC) denoting restricted movement of water molecules and high cellularity. The red box corresponds to the zoomed area represented in the ROI column. The red circle represents the ROI, and the white box represents the measured mADC value. ROI: region of interest.

MRI images were acquired in the axial plane and included T1-weighted without contrast, T2-weighted, DWI and ADC maps. All ADC maps were analyzed using the Arya Pixeon Medical System program (Pixeon). Restricted diffusion of water molecules and high cellularity were evaluated in the section that exhibited the greatest restricted diffusion represented by the largest hypersignal area in DWI, confirmed by the loss of the corresponding image signal in the ADC map. Accordingly, two experienced radiologists (CEBC and FMPS) determined the lowest (darkest part of image) ADC value to draw a 6 mm^2^ circular region of interest (ROI) completely within the tumor, avoiding areas of necrosis, hemorrhage, adipose tissue, cystic tissue, and noisy or nonenhancing lesions, supported by the correlation with T1- and T2-weighted sequences (Fig 1). For other tumors, it is known that the selection of the lowest ADC value within the lesion (corresponding to the most active part of the lesion) might provide a more accurate discrimination between malignant and benign lesions [22, 23]. Additionally, the strategy described above is suggested as the preferred method for measuring ADC values to reduce inter- and intrareader variability and improve DWI consistency and comparability between sites in other tumors [23]. ADC values were obtained using two values of b (b = 50 mm^2^/s or b = 800 mm^2^/s) and reported in units of 10^−3^ mm^2^/s. The acquisition parameters used for MRI images are demonstrated in S2 Table.

### Statistical analysis

Descriptive statistics (mean, standard deviation medians and proportions) were used to characterize the clinical and demographic data. The relationship between ADC and histological subtype was assessed using ANOVA, and multiple comparisons were performed by the Bonferroni correction. Normality of distribution for the variables was determined by the Shapiro–Wilk test. Relations were assessed using the Chi-square or Fisher’s exact test as appropriate for categorical variables. Receiver operating characteristic (ROC) curve analysis and area under the curve (AUC) were used to determine optimal cutoff values of ADC and to determine its potential diagnostic performance. For all analyses, values of p < 0.05 were considered indicative of a significant difference. Statistical analysis was performed using IBM SPSS version 27.0 software (SPSS, Chicago, IL, USA).

## Results

We evaluated MRI images from primary tumors at diagnosis of 43 pediatric patients confirmed with germ cell tumors (gonadal or extragonadal). The demographic and clinicopathological characteristics of the patients are shown in Table 1.

**Table 1:** Demographic and clinicopathological characteristics of the 43 pediatric patients diagnosed with primary germ cell tumors (gonadal or extracranial) included in the study.

Demographic and clinicopathological characteristics		(n = 43)	%
Age (years; 11.17 ± 5.33)			
Sex	Female	30	69.8
	Male	13	30.2
Tumor histology	Pure teratoma	14	32.6
	Yolk sac	9	20.9
	Immature teratoma	5	11.6
	Dysgerminoma	5	11.6
	Mixed teratoma^a^	4	9.3
	Mixed GCT^b^	6	14.0
Histology classification	Malignant	24	55.8
	Benign	14	32.6
	Immature teratoma	5	11.6
Tumor staging	I	24	57.1
	II	0	-
	III	10	23.8
	IV	8	19.1
Patient status	Alive (no disease)^c^	31	72.1
	Alive (in treatment)^d^	12	27.9

^**a**^ Mixed teratoma: teratoma is one of the components of mixed tumor;

^**b**^ Mixed GCT: no teratoma component in mixed tumor;

^**c**^ At the time of the study, patients were alive without disease;

^**d**^ At the time of the study, patients were alive and receiving treatment;

BGC-PED-Study Group: Brazilian Germ Cell Pediatric Study Group

The normality of the ADC variable was verified by the Shapiro–Wilk test in each histology category (Table 2).

**Table 2:** Normality distribution of ADC values evaluated by the Shapiro–Wilk test.

Histology	P value
Pure teratoma	0.870
Immature teratoma	0.535
Yolk Sac	0.165
Mixed teratoma	0.126
Dysgerminoma	0.698
Mixed GCT	0.832

Mean ADC (mADC) values for the different GCT histologies are demonstrated in Table 3. Our findings demonstrated an increased mADC value in pure teratomas (1,403.50 ± 161.76 x10^-3^ mm^2^/s; mean ± SD) with a statistically significant difference when compared to the other histologies of GCT (Table 3). Dysgerminomas presented the lowest mADC value (646.00 ± 65.18 x10^-3^ mm^2^/s; mean ± SD) in our cohort; however, excluding pure teratomas, no statistically significant difference was observed compared to the other GCT histologies.

**Table 3:** Mean ADC values for the GCT histology types from the primary tumors of the 43 patients included in the study.

Histology	Mean ADC (x10^-3^ mm^2^/s)					
	mean	SD	median	min.	max.	p value*
Pure teratoma	1,403.50	161.76	1,398.00	1,081.00	1,669.00	-
Immature teratoma	1,052.20	314.15	1,058.00	631.00	1,382.00	0.034
Yolk sac	859.78	256.02	840.00	603.00	1,383.00	< 0.01
Mixed teratoma^a^	809.75	232.17	723.00	650.00	1,143.00	< 0.01
Dysgerminoma	646.00	65.18	655.00	544.00	715.00	< 0.01
Mixed GCT^b^	733.67	160.96	705.00	525.00	952.00	< 0.01

*P value: determined by comparing pure teratoma histology with the other histologies of GCT using the Bonferroni statistical test.

^**a**^ Mixed teratoma: teratoma is one of the components of mixed tumor;

^**b**^ Mixed GCT: no teratoma component in mixed tumor;

SD: standard deviation; min: minimum; max: maximum

**Fig 2:**
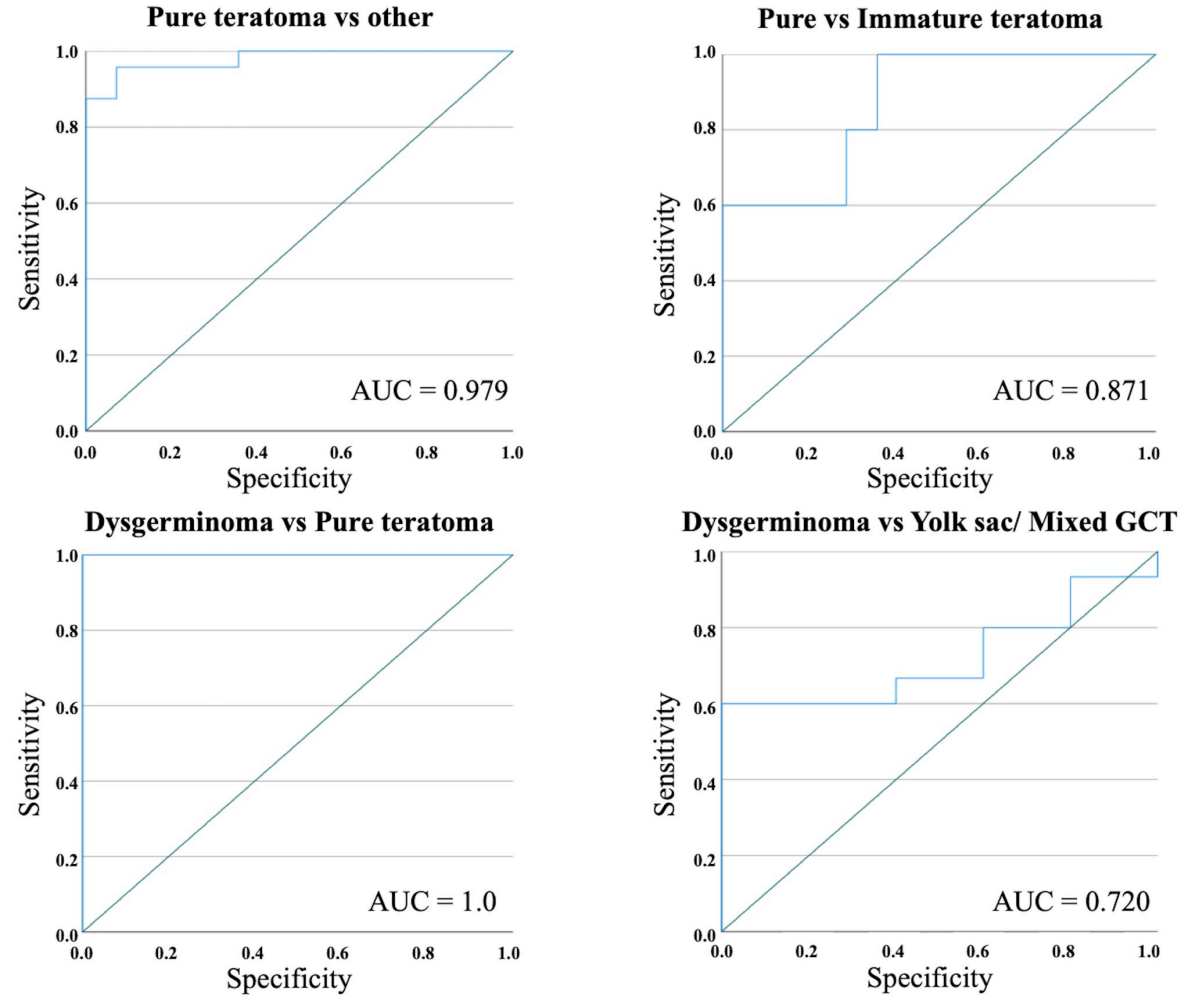
Receiver operating characteristic curve (ROC) analysis. ROC analysis for prediction of pediatric extracranial germ cell tumor types based on mADC value using diffusion-weighted imaging in magnetic resonance. AUC (area under the curve).

Excluding immature teratomas, we performed an ROC analysis considering mADC and determined a cutoff value of 1,179.00 x 10^−3^ mm^2^/s that differentiated pure teratomas from the other histologies (yolk sac, mixed teratoma, dysgerminoma and mixed GCT) of GCT with a sensitivity of 95.8% and a specificity of 92.9% (AUC = 0.979; p<0.01) if pure teratoma was predicted to have a higher mADC value than the cutoff (Fig 2). Considering teratomas, we determined a cutoff value of 1,337.00 x 10^−3^ mm^2^/s that differentiated pure from immature histology with a sensitivity of 80.0% and a specificity of 71.4% (AUC = 0.871; p = 0.016) if pure teratomas were predicted to have a higher mADC value than the cutoff (Fig 2). Additionally, considering dysgerminomas, we determined a cutoff value of 898.0 x 10^−3^ mm^2^/s that differentiated dysgerminomas from benign GCTs (pure teratoma) with a sensitivity of 100.0% and a specificity of 100.0% (AUC = 1.0; p<0.01) if dysgerminoma was predicted have a lower mADC value than the cutoff (Fig 2). Regarding malignant disease (dysgerminoma, yolk sac and mixed GCTs), ROC analysis was not able to establish a significant cutoff mADC value with acceptable sensitivity and specificity between these histologies (Fig 2).

**Fig 3:**
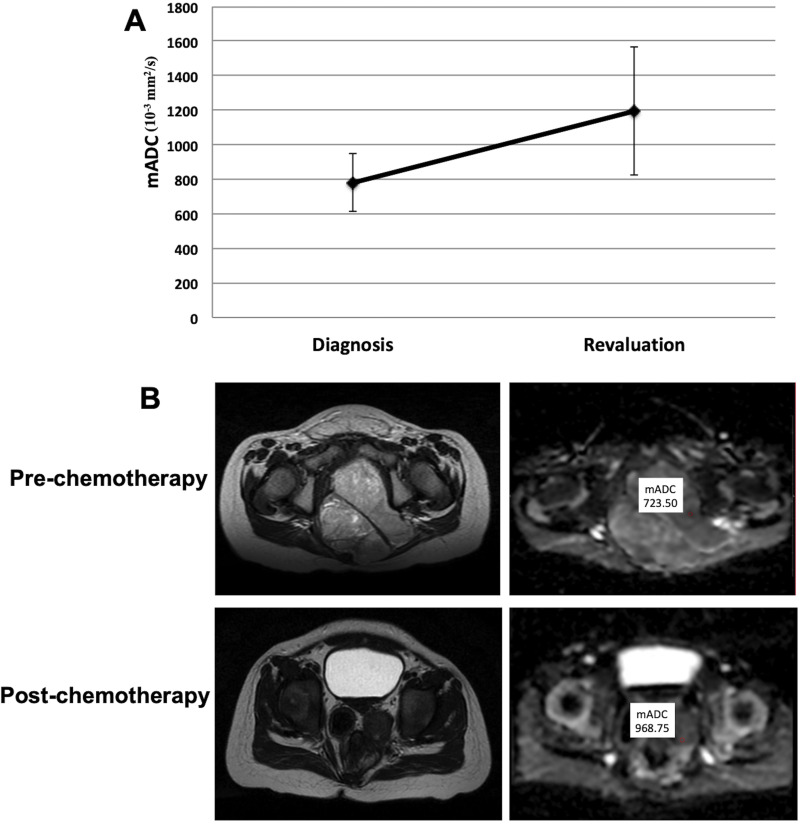
mADC measurements of malignant GCT patients at diagnosis and during treatment. A. mADC was determined for 11 patients with malignant GCT at diagnosis, revaluated during treatment, and compared using the generalized linear statistical model. B. T2w and ADC maps of one representative patient with yolk sak tumor pre and post chemotherapy. The red circle represents the ROI, and the white box represents the measured mADC value. ROI: region of interest.

Additionally, we evaluated mADC values for 11 patients with malignant GCT with DWI images available at diagnosis and in revaluation during treatment using the generalized linear statistical model. We observed that compared to the diagnosis (780.00 ± 168.00 mm^2^/s; mean ± SD), we observed a significant increase in mADC when evaluating patients during treatment (1,197.00 ± 372.00 mm^2^/s; p<0.001) (Fig 3). The majority of the patients underwent surgery for tumor resection to consolidate treatment, which made it impossible to analyze tumor images at the end of the treatment.

## Discussion

Recent studies have shown that the high capacity to differentiate tissues and collect biochemical information makes MRI the most efficient diagnostic imaging method for evaluating GCT at its primary site. MRI presenting T1- and T2-weighted sequences provides details of soft tissues at a higher level of discrimination than computed tomography (CT). The addition of the diffusion sequence of free water molecules does not significantly increase the examination time and does not require intravenous paramagnetic contrast. Higher tissue cellularity or increased cell density results in low ADC values [14]. However, ADC measurements as a tool to support tumor diagnosis and/or response to treatment are still not known in pediatric germ cell tumors [24].

In our study, we demonstrated that mADC values could be useful for distinguishing malignant from benign origins of pediatric GCTs, but more specifically, it can be used to discriminate pure teratomas from GCTs of other histologies at diagnosis. We showed that decreased mADC values (1,179.00 x 10^−3^ mm^2^/s) were associated with malignant tumors, indicating an increase in cell density that is characteristic of this group of tumors (ROC AUC = 0.979; p<0.01). These findings were significantly more evident in dysgerminomas due to the presence of higher tumor cellularity or increased tumor cell density (lowest mADC values). Thus, MRI with diffusion can be used as an important tool in the evaluation of tumor cellularity and density and the differential diagnosis of dysgerminomas or pure teratomas.

A report evaluating DWI and ADC maps in pediatric intracranial GCTs also indicated that the mADC of solid tumor portions was significantly lower in the germinoma group (1.113 ± 0.415 x 10^−3^ mm^2^/s) than in the nongerminomatous GCT (NGGCT) group (1.499 ± 0.469 x 10^−3^ mm^2^/s, p = 0.001). Additionally, the authors demonstrated that the mADC threshold value (1.143 x 10^−3^ mm^2^/s) had the highest specificity (91.3%) and positive predictive value (92.3%) for discriminating germinomas from NGGCTs [25]. These data corroborate our observations in pediatric extracranial germ cell tumors.

Recently, MRI with a diffusion sequence of water molecules was shown to be sensitive to changes in the tumor cell density represented by significant damage to tumor cells related to the breakdown of the membrane barrier and reduction of tumor cellularity after chemotherapy [26]. Patients with malignant GCT who have no surgical indication are treated with chemotherapy. Therapeutic response prediction is possible in GCTs that express biological markers (AFP, BHCG and LDH); however, in some histological GCT subtypes, such biological markers are not expressed [27].

We demonstrated, using MRI with diffusion, that at least some of the tumors evaluated at diagnosis (without treatment) showed an increased mADC value at the moment of revaluation during treatment, which could reflect a therapeutic response with treatment-induced cell death. In this context, some scholars have reported the association of ADC with short-term clinical outcomes in other types of cancer lesions, such as the early response to chemotherapy [28, 29]. However, the observation of ADC at different time points among different patients in our study limits our understanding of its potential role in monitoring a patient’s therapeutic response. More patients should be evaluated in such a context to confirm whether mADC increases during treatment could be used together with serum markers as a parameter to monitor treatment response.

The relatively small number of cases evaluated and the retrospective design of the study could represent limiting factors in data interpretation. Additionally, another limitation is associated with data (b-values) generated using different 1.5 T scanners. However, scholars reported in one study that changing b-value combinations for ADC analysis had no impact on the discrimination between benign and malignant lesions [30].

## Conclusions

Together, our data suggest that mADC evaluation at GCT diagnosis can be used as a support tool for the discrimination of benign (pure teratoma) from malignant tumors (dysgerminoma, yolk sac and mixed GCT). Additionally, our study showed evidence that mADC could also be used as a complementary tool to monitor treatment response in patients with malignant GCT. Due to the heterogeneity of the histological GCT subtypes as well as their lower occurrence in children and adolescents, we consider that the assessment of ADC as a predictor of response and clinical outcomes needs to be validated in a larger cohort and a prospective scenario.

## Supporting information

S1 Table Tumor histology, primary site, surgery/biopsy and tumor size of the of the 43 pediatric patients diagnosed with primary germ cell tumors (gonadal or extracranial) included in the study. (PDF)

S2 Table The acquisition parameters used for MRI images. (PDF)

S3 Table Raw data. (PDF)

## References

[1] Schneider DT, Calaminus G, Koch S, Teske C, Schmidt P, Haas RJ, et al. Epidemiologic analysis of 1,442 children and adolescents registered in the German germ cell tumor protocols. Pediatr Blood Cancer. 2004. pp. 169–75. doi: 10.1002/pbc.10321 14752882

[2] Lopes LF, Macedo CR, Pontes EM, Dos Santos Aguiar S, Mastellaro MJ, Melaragno R, et al. Cisplatin and etoposide in childhood germ cell tumor: brazilian pediatric oncology society protocol GCT-91. J Clin Oncol. 2009. pp. 1297–303. doi: 10.1200/JCO.2008.16.4202 19164215

[3] Lopes LF, Sonaglio V, Ribeiro KC, Schneider DT, de Camargo B. Improvement in the outcome of children with germ cell tumors. Pediatr Blood Cancer. 2008. pp. 250–3. doi: 10.1002/pbc.21268 17554793

[4] Faure Conter C, Xia C, Gershenson D, Hurteau J, Covens A, Pashankar F, et al. Ovarian Yolk Sac Tumors; Does Age Matter? Int J Gynecol Cancer. 2018;28: 77–84. doi: 10.1097/IGC.0000000000001149 29194189

[5] Pierce JL, Frazier AL, Amatruda JF. Pediatric Germ Cell Tumors: A Developmental Perspective. Adv Urol. 2018;2018: 9059382. doi: 10.1155/2018/9059382 29515628 PMC5817207

[6] Fonseca A, Frazier AL, Shaikh F. Germ Cell Tumors in Adolescents and Young Adults. J Oncol Pract. 2019;15: 433–441. doi: 10.1200/JOP.19.00190 31404512

[7] Moch H, Cubilla AL, Humphrey PA, Reuter VE, Ulbright TM. The 2016 WHO Classification of Tumours of the Urinary System and Male Genital Organs-Part A: Renal, Penile, and Testicular Tumours. Eur Urol. 2016. pp. 93–105. doi: 10.1016/j.eururo.2016.02.029 26935559

[8] Göbel U, Calaminus G, Engert J, Kaatsch P, Gadner H, Bökkerink JP, et al. Teratomas in infancy and childhood. Med Pediatr Oncol. 1998;31: 8–15. doi: 10.1002/(sici)1096-911x(199807)31:1&lt;8::aid-mpo2&gt;3.0.co;2-h 9607423

[9] Lorch A, Bascoul-Mollevi C, Kramar A, Einhorn L, Necchi A, Massard C, et al. Conventional-dose versus high-dose chemotherapy as first salvage treatment in male patients with metastatic germ cell tumors: evidence from a large international database. J Clin Oncol. 2011. pp. 2178–84. doi: 10.1200/JCO.2010.32.6678 21444870

[10] Hayes C, Padhani AR, Leach MO. Assessing changes in tumour vascular function using dynamic contrast-enhanced magnetic resonance imaging. NMR Biomed. 2002. pp. 154–63. doi: 10.1002/nbm.756 11870911

[11] Pickles MD, Gibbs P, Lowry M, Turnbull LW. Diffusion changes precede size reduction in neoadjuvant treatment of breast cancer. Magn Reson Imaging. 2006. pp. 843–7. doi: 10.1016/j.mri.2005.11.005 16916701

[12] Britten RA, Evans AJ, Allalunis-Turner MJ, Franko AJ, Pearcey RG. Intratumoral heterogeneity as a confounding factor in clonogenic assays for tumour radioresponsiveness. Radiother Oncol. 1996;39: 145–153. doi: 10.1016/0167-8140(96)01719-7 8735482

[13] Gilligan TD, Seidenfeld J, Basch EM, Einhorn LH, Fancher T, Smith DC, et al. American Society of Clinical Oncology Clinical Practice Guideline on uses of serum tumor markers in adult males with germ cell tumors. J Clin Oncol. 2010;28: 3388–3404. doi: 10.1200/JCO.2009.26.4481 20530278

[14] Padhani AR, Liu G, Koh DM, Chenevert TL, Thoeny HC, Takahara T, et al. Diffusion-weighted magnetic resonance imaging as a cancer biomarker: consensus and recommendations. Neoplasia. 2009. pp. 102–25. doi: 10.1593/neo.81328 19186405 PMC2631136

[15] Lee KC, Moffat BA, Schott AF, Layman R, Ellingworth S, Juliar R, et al. Prospective early response imaging biomarker for neoadjuvant breast cancer chemotherapy. Clin Cancer Res. 2007. pp. 443–50. doi: 10.1158/1078-0432.CCR-06-1888 17255264

[16] Sinkus R, Van Beers BE, Vilgrain V, DeSouza N, Waterton JC. Apparent diffusion coefficient from magnetic resonance imaging as a biomarker in oncology drug development. Eur J Cancer. 2012. pp. 425–31. doi: 10.1016/j.ejca.2011.11.034 22226479

[17] Padhani AR. Diffusion magnetic resonance imaging in cancer patient management. Semin Radiat Oncol. 2011. pp. 119–40. doi: 10.1016/j.semradonc.2010.10.004 21356480

[18] Naganawa S, Sato C, Kumada H, Ishigaki T, Miura S, Takizawa O. Apparent diffusion coefficient in cervical cancer of the uterus: comparison with the normal uterine cervix. Eur Radiol. 2005. pp. 71–8. doi: 10.1007/s00330-004-2529-4 15538578

[19] Min X, Feng Z, Wang L, Cai J, Yan X, Li B, et al. Characterization of testicular germ cell tumors: Whole-lesion histogram analysis of the apparent diffusion coefficient at 3T. Eur J Radiol. 2018. pp. 25–31. doi: 10.1016/j.ejrad.2017.10.030 29279166

[20] Pereira A, Pérez-Medina T, Magrina JF, Magtibay PM, Rodríguez-Tapia A, Peregrin I, et al. International Federation of gynecology and obstetrics staging classification for cancer of the ovary, fallopian tube, and peritoneum: estimation of survival in patients with node-positive epithelial ovarian cancer. Int J Gynecol Cancer. 2015. pp. 49–54. doi: 10.1097/IGC.0000000000000316 25405578

[21] Olson TA, Murray MJ, Rodriguez-Galindo C, Nicholson JC, Billmire DF, Krailo MD, et al. Pediatric and Adolescent Extracranial Germ Cell Tumors: The Road to Collaboration. J Clin Oncol. 2015;33: 3018–3028. doi: 10.1200/JCO.2014.60.5337 26304902 PMC4979195

[22] Arponen O, Sudah M, Masarwah A, Taina M, Rautiainen S, Könönen M, et al. Diffusion-Weighted Imaging in 3.0 Tesla Breast MRI: Diagnostic Performance and Tumor Characterization Using Small Subregions vs. Whole Tumor Regions of Interest. PLoS One. 2015;10: e0138702. doi: 10.1371/journal.pone.0138702 26458106 PMC4601774

[23] Baltzer P, Mann RM, Iima M, Sigmund EE, Clauser P, Gilbert FJ, et al. Diffusion-weighted imaging of the breast-a consensus and mission statement from the EUSOBI International Breast Diffusion-Weighted Imaging working group. Eur Radiol. 2020;30: 1436–1450. doi: 10.1007/s00330-019-06510-3 31786616 PMC7033067

[24] Tsuboyama T, Hori Y, Hori M, Onishi H, Tatsumi M, Sakane M, et al. Imaging findings of ovarian dysgerminoma with emphasis on multiplicity and vascular architecture: pathogenic implications. Abdom Radiol (NY). 2018;43: 1515–1523. doi: 10.1007/s00261-018-1503-6 29450608

[25] Wu C-C, Guo W-Y, Chang F-C, Luo C-B, Lee H-J, Chen Y-W, et al. MRI features of pediatric intracranial germ cell tumor subtypes. J Neurooncol. 2017;134: 221–230. doi: 10.1007/s11060-017-2513-x 28551848

[26] Marconi DG, Fregnani JHTG, Rossini RR, Netto AKBJ, Lucchesi FR, Tsunoda AT, et al. Pre-treatment MRI minimum apparent diffusion coefficient value is a potential prognostic imaging biomarker in cervical cancer patients treated with definitive chemoradiation. BMC Cancer. 2016;16: 556. doi: 10.1186/s12885-016-2619-0 27469349 PMC4965898

[27] Drozynska E, Bien E, Polczynska K, Stefanowicz J, Zalewska-Szewczyk B, Izycka-Swieszewska E, et al. A need for cautious interpretation of elevated serum germ cell tumor markers in children. Review and own experiences. Biomark Med. 2015;9: 923–932. doi: 10.2217/bmm.15.42 26329804

[28] Cui Y, Zhang X-P, Sun Y-S, Tang L, Shen L. Apparent diffusion coefficient: potential imaging biomarker for prediction and early detection of response to chemotherapy in hepatic metastases. Radiology. 2008;248: 894–900. doi: 10.1148/radiol.2483071407 18710982

[29] Heijmen L, Ter Voert EEGW, Nagtegaal ID, Span P, Bussink J, Punt CJA, et al. Diffusion-weighted MR imaging in liver metastases of colorectal cancer: reproducibility and biological validation. Eur Radiol. 2013;23: 748–756. doi: 10.1007/s00330-012-2654-4 23001604

[30] Hoogendam JP, Klerkx WM, de Kort GAP, Bipat S, Zweemer RP, Sie-Go DMDS, et al. The influence of the b-value combination on apparent diffusion coefficient based differentiation between malignant and benign tissue in cervical cancer. J Magn Reson Imaging. 2010;32: 376–382. doi: 10.1002/jmri.22236 20677265

