# Supplementary material for: Diffusion-weighted imaging in pediatric extracranial germ cell tumors
Source: PLoS One. 2023 Nov 30;18(11):e0294976. doi: 10.1371/journal.pone.0294976 (PMC10688858; doi:10.1371/journal.pone.0294976)
Supplement: S1 Table — (PDF) [file pone.0294976.s001.pdf]

S1 Table. Tumor histology, primary site, surgery/biopsy and tumor size of the 43 pediatric patients diagnosed with primary germ cell tumors (gonadal or extracranial) included in the study.

					**Tumor size (centimeter)**		
**Sample ID**	**Histology**	**Primary site**	**Surgery**	**Biopsy**	**AP**	**TRV**	**CC**
1	Yolk Sac	Retroperitoneum	diagnosis	No	-	-	-
2	Yolk Sac	Head and neck	diagnosis and in the end of treatment	Yes	8.7	7	12
3	Yolk Sac	Sacrococcygeal	end of treatment	Yes	8	7.5	10.5
4	Yolk Sac	Ovary	end of treatment	Yes	31.1	14.6	20.5
5	Yolk Sac	Ovary	diagnosis and in the end of treatment	No	15.5	11.8	8.1
6	Yolk Sac	Ovary	end of treatment	Yes	15	20.5	24
7	Yolk Sac	Sacrococcygeal	end of treatment	No	5.8	6	8
8	Yolk Sac	Ovary	diagnosis	No	9,3	15,7	16,5
9	Yolk Sac	Testis	diagnosis	No	11.8	10.6	9.3
10	Dysgerminoma	Ovary	diagnosis	No	-	-	-
11	Dysgerminoma	Ovary	diagnosis	No	29	12.4	19.5
12	Dysgerminoma	Ovary	diagnosis	No	9.5	18.6	20
13	Dysgerminoma	Ovary	diagnosis and in the end of treatment	No	-	-	-
14	Dysgerminoma	Ovary	end of treatment	Yes	5.6	7.4	11.1
15	Mixed GCT	Testis	diagnosis	No	-	-	-
16	Mixed GCT	Ovary	diagnosis	No	7.5	6	8.8
17	Mixed GCT	Ovary	end of treatment	Yes	11	17	19
18	Mixed GCT	Testis	end of treatment	Yes	10	9.7	8.5
19	Mixed GCT	Ovary	end of treatment	No	30.6	14	21.5
20	Mixed GCT	Testis	diagnosis	No	3.6	5.8	8
21	Pure teratoma	Ovary	diagnosis	No	-	-	-
22	Pure teratoma	Ovary	diagnosis	No	-	-	-
23	Pure teratoma	Ovary	diagnosis	No	-	-	-
24	Pure teratoma	Ovary	diagnosis	No	-	-	-
25	Pure teratoma	Ovary	diagnosis	No	-	-	-
26	Pure teratoma	Ovary	diagnosis	No	15.7	21.3	20.4
27	Pure teratoma	Sacrococcygeal	diagnosis	No	4.9	6.3	8.5
28	Pure teratoma	Ovary	diagnosis	No	4	3.5	4.5
29	Pure teratoma	Sacrococcygeal	diagnosis	No	7.5	11.5	15
30	Pure teratoma	Ovary	diagnosis	No	5,3		3,5
31	Pure teratoma	Ovary	diagnosis	No	2.5		2
32	Pure teratoma	Ovary	diagnosis	No	9.7	8.7	7.8
33	Pure teratoma	Testis	diagnosis	No	2		1.8
34	Pure teratoma	Ovary	diagnosis	No	13.4	21	32
35	Immature teratoma	Sacrococcygeal	diagnosis	No	-	-	-
36	Immature teratoma	Ovary	diagnosis	No	6.8	11	10.9
37	Immature teratoma	Retroperitoneum	diagnosis	No	9.8	11.1	11.6
38	Immature teratoma	Sacrococcygeal	diagnosis	No	8.2	13.6	16
39	Immature teratoma	Retroperitoneum	diagnosis	No	-	-	-
40	Mixed teratoma	Testis	diagnosis and after three cycles of chemotherapy	No	1.3	.9	
41	Mixed teratoma	Ovary	diagnosis and in the end of treatment	No	-	-	-
42	Mixed teratoma	Mediastinum	end of treatment	Yes	-	-	-
43	Mixed teratoma	Testis	diagnosis	No	8.7	7.4	6.5

AP: anteroposterior TRV: transversal CC: craniocaudal
