# Supplementary material for: Diffusion-weighted imaging in pediatric extracranial germ cell tumors
Source: PLoS One. 2023 Nov 30;18(11):e0294976. doi: 10.1371/journal.pone.0294976 (PMC10688858; doi:10.1371/journal.pone.0294976)
Supplement: S2 Table — (PDF) [file pone.0294976.s002.pdf]

S2 Table. The acquisition parameters used for MRI images.

Sequence	DIFFUSION B 50	Sequence	AXIAL T2 TE 80
Repetition time, ms	12000	Repetition time, ms	12000
Echo time, ms	Minimum	Echo time, ms	80
Voxelsize, mm3		Voxelsize, mm3	
Field of view (read x phase) mm2	128x128	Field of view (read x phase) mm2	320x224
Phase direction	128	Phase direction	320
Phase resolution	128	Phase resolution	224
Partial Fourier		Partial Fourier	
Matrix		Matrix	320x224
Slice distence	5.0	Slice distence	0
Nº slices	42	Nº slices	50
Parallel imaging		Parallel imaging	
Bandwidthh, Hz/pixel	25	Bandwidthh, Hz/pixel	41.67
Echospacing, ms		Echospacing, ms	
b-values, s/mm²	50	b-values, s/mm²	no
Averags (b50,b800)	12	Averags (b50,b800)	no
Diffusion mode		Diffusion mode	no
Diffusion scheme		Diffusion scheme	no
Acqisition min		Acqisition min	
Trigger	yes	Trigger	yes
Prescan normalize	yes	Prescan normalize	yes
fat saturation	no	fat saturation	yes

Sequence	Dynamic 3D LAVA
Repetition time, ms	4.4
Echo time, ms	1.8
Voxelsize, mm3	
Field of view (read x phase) mm2	256x192
Phase direction	256
Phase resolution	192
Partial Fourier	
Matrix	
Slice distence	0.4
Nº slices	58
Parallel imaging	
Bandwidthh, Hz/pixel	62.5
Echospacing, ms	
b-values, s/mm²	no
Averags (b50,b800)	no
Diffusion mode	no
Diffusion scheme	no
Acqisition min	no
Trigger	no
Prescan normalize	
fat saturation	Special

Sequence	AXIAL T2 FINE
Repetition time, ms	4300
Echo time, ms	60
Voxelsize, mm3	
Field of view (read x phase) mm2	288x224
Phase direction	288
Phase resolution	
Partial Fourier	224
Matrix	
Slice distence	3.0
Nº slices	44
Parallel imaging	
Bandwidthh, Hz/pixel	35.71
Echospacing, ms	
b-values, s/mm²	no
Averags (b50,b800)	no
Diffusion mode	no
Diffusion scheme	no
Acqisition min	
Trigger	yes
Prescan normalize	
fat saturation	no

Sequence	AXIAL IN/OUT FASE
Repetition time, ms	Minimum
Echo time, ms	2
Voxelsize, mm3	
Field of view (read x phase) mm2	
Phase direction	
Phase resolution	288
Partial Fourier	224
Matrix	288x224
Slice distence	0
Nº slices	26
Parallel imaging	
Bandwidthh, Hz/pixel	62.5
Echospacing, ms	
b-values, s/mm²	no
Averags (b50,b800)	no
Diffusion mode	no
Diffusion scheme	no
Acqisition min	
Trigger	no
Prescan normalize	Yes
fat saturation	no

Sequence	CORONAL T2
Repetition time, ms	Minimum
Echo time, ms	110
Voxelsize, mm3	
Field of view (read x phase) mm2	320x224
Phase direction	320
Phase resolution	224
Partial Fourier	
Matrix	
Slice distence	0
Nº slices	34
Parallel imaging	
Bandwidthh, Hz/pixel	41.67
Echospacing, ms	
b-values, s/mm²	no
Averags (b50,b800)	no
Diffusion mode	no
Diffusion scheme	no
Acqisition min	
Trigger	no
Prescan normalize	yes
fat saturation	no

Sequence	CORONAL T2 FINE
Repetition time, ms	4200
Echo time, ms	60
Voxelsize, mm3	
Field of view (read x phase) mm2	288x224
Phase direction	288
Phase resolution	224
Partial Fourier	
Matrix	
Slice distence	3.0
Nº slices	26
Parallel imaging	
Bandwidthh, Hz/pixel	41.67
Echospacing, ms	
b-values, s/mm²	no
Averags (b50,b800)	no
Diffusion mode	no
Diffusion scheme	no
Acqisition min	
Trigger	yes
Prescan normalize	yes
fat saturation	no
