# Supplementary material for: Diffusion-weighted imaging in pediatric extracranial germ cell tumors
Source: PLoS One. 2023 Nov 30;18(11):e0294976. doi: 10.1371/journal.pone.0294976 (PMC10688858; doi:10.1371/journal.pone.0294976)
Supplement: S3 Table — (PDF) [file pone.0294976.s003.pdf]

S3 Table. Raw data.

Sample_ID	Gender	Age (diagnosis_months)	Tumor_primary_site	Tumor_staging	Tumor_histology	Minimum_ADC_pretreatment(diagnosis)
1	Female	217,87	Head	IV	Yolk Sac	545,00
2	Female	165,34	Head and neck	III	Yolk Sac	299,00
3	Female	43,69	Sacrococcygeal	III	Yolk Sac	682,00
4	Female	116,29	Ovary	IV	Yolk Sac	679,00
5	Female	223,98	Ovary	I	Yolk Sac	1.328,00
6	Female	159,20	Ovary	III	Yolk Sac	
7	Female	14,42	Sacrococcygeal	IV	Yolk Sac	
8	Female		Ovary	III	Yolk Sac	
9	Male		Testis	-	Yolk Sac	
10	Female	57,79	Ovary	III	Dysgerminoma	459,00
11	Female	199,38	Ovary	I	Dysgerminoma	549,00
12	Female	162,09	Ovary	I	Dysgerminoma	577,00
13	Female	160,38	Ovary	III	Dysgerminoma	470,00
14	Female	95,07	Ovary	III	Dysgerminoma	
15	Male	199,28	Testis	IV	Mixed GCT	748,00
16	Female	35,78	Ovary	I	Mixed GCT	461,00
17	Female	118,79	Ovary	IV	Mixed GCT	
18	Male	209,63	Testis	IV	Mixed GCT	
19	Female	176,22	Ovary	III	Mixed GCT	
20	Male	189,16	Testis	IV	Mixed GCT	
21	Female	183,31	Ovary	I	Pure teratoma	659,00
22	Female	187,58	Ovary	I	Pure teratoma	574,00
23	Female	108,94	Ovary	I	Pure teratoma	943,00
24	Female	133,94	Ovary	I	Pure teratoma	1.154,00
25	Female	240,77	Ovary	I	Pure teratoma	957,00
26	Female	180,91	Ovary	I	Pure teratoma	1.062,00
27	Male	4,50	Sacrococcygeal	I	Pure teratoma	897,00
28	Female	161,83	Ovary	I	Pure teratoma	
29	Female	0,07	Sacrococcygeal	I	Pure teratoma	
30	Female	174,34	Ovary	I	Pure teratoma	
31	Female	214,45	Ovary	I	Pure teratoma	

Sample_ID	Gender	Age (diagnosis_months)	Tumor_primary_site	Tumor_staging	Tumor_histology	Minimum_ADC_pretreatment(diagnosis)
32	Female	186,56	Ovary	I	Pure teratoma	
33	Male	40,51	Testis	I	Pure teratoma	
34	Female		Ovary	I	Pure teratoma	
35	Male	0,13	Sacrococcygeal	I	Immature teratoma	657,00
36	Female	112,12	Ovary	I	Immature teratoma	1.150,00
37	Male	5,75	Retroperitoneum	I	Immature teratoma	
38	Female		Sacrococcygeal	I	Immature teratoma	
39	Male	0,56	Retroperitoneum	I	Immature teratoma	
40	Male	191,72	Testis	IV	Mixed teratoma	810,00
41	Female	211,34	Ovary	III	Mixed teratoma	970,00
42	Male	136,07	Mediastinum	III	Mixed teratoma	451,00
43	Male	207,75	Testis	I	Mixed teratoma	669,00

Mean_ADC_pretreatment(diagnosis)	Maximum_ADC_pretreatment(diagnosis)	Standard_deviation_ADC_pretreatment(diagnosis)
840,00	1.396,00	169,00
603,00	1.078,00	146,00
641,00	995,00	62,00
898,00	1.131,00	116,00
1.383,00	1.828,00	29,00
765,00		
848,00		
1.130,00		
630,00		
544,00	1.113,00	87,00
631,00	859,00	53,00
715,00	927,00	93,00
685,00	898,00	73,00
655,00		
952,00	836,00	29,00
525,00	709,00	44,00
750,00		
660,00		
632,00		
883,00		
1.553,00	1.579,00	190,00
1.399,00	2.863,00	349,00
1.081,00	1.815,00	178,00
1.396,00	1.794,00	157,00
1.591,00	2.251,00	253,00
1.268,00	1.252,00	49,00
1.591,00	1.296,00	81,00
1.436,00		
1.397,00		
1.669,00		
1.282,00		

Mean_ADC_pretreatment(diagnosis)	Maximum_ADC_pretreatment(diagnosis)	Standard_deviation_ADC_pretreatment(diagnosis)
1.350,00		
1.215,00		
1.421,00		
1.058,00	1.147,00	193,00
1.382,00	2.457,00	474,00
1.324,00		
866,00		
631,00		
794,00	1.267,00	123,00
1.143,00	1.776,00	160,00
652,00	835,00	100,00
650,00	818,00	44,00

Minimum_ADC_reevaluation	Mean_ADC_reevaluation	Maximum_ADC_reevaluation	Standard_deviation_ADC_reevaluation	Patient_status
				Alive (no disease)
1.012,00	1.646,00	2.078,00	298,00	Alive (in treatment)
164,00	1.249,00	1.372,00	50,00	Alive (in treatment)
913,00	1.044,00	1.233,00	93,00	Alive (in treatment)
				Alive (no disease)
				Alive (in treatment)
	1.098,00			Alive (no disease)
				Alive (no disease)
				Alive (in treatment)
				Alive (no disease)
				Alive (no disease)
				Alive (no disease)
				Alive (in treatment)
	1.405,00			Alive (in treatment)
998,00	1.177,00	1.458,00	129,00	Alive (in treatment)
				Alive (no disease)
	698,00			Alive (no disease)
	805,00			Alive (no disease)
	784,00			Alive (no disease)
				Alive (in treatment)
				Alive (no disease)
				Alive (no disease)
				Alive (no disease)
				Alive (no disease)
				Alive (no disease)
				Alive (no disease)
				Alive (no disease)
				Alive (no disease)
				Alive (no disease)
				Alive (no disease)
				Alive (no disease)

Minimum_ADC_reevaluation	Mean_ADC_reevaluation	Maximum_ADC_reevaluation	Standard_deviation_ADC_reevaluation	Patient_status
				Alive (no disease)
				Alive (no disease)
				Alive (no disease)
				Alive (no disease)
				Alive (no disease)
				Alive (no disease)
				Alive (no disease)
				Alive (no disease)
1.147,00	1.353,00	1.584,00	99,00	Alive (in treatment)
1.160,00	1.908,00	2.527,00	333,00	Alive (in treatment)
				Alive (in treatment)
				Alive (no disease)

			Tumor size (centimeter)		
**Age_(last_information_or_death)**	**Surgery**	**Biopsy**	**AP**	**TRV**	**CC**
246,42	diagnosis	No	-	-	-
175,72	diagnosis and in the end of treatment	Yes	8.7	7,00	12,00
55,95	end of treatment	Yes	8,00	7.5	10.5
127,79	end of treatment	Yes	31.1	14.6	20.5
234,46	diagnosis and in the end of treatment	No	15.5	11.8	8.1
162,71	end of treatment	Yes	15,00	20.5	24,00
36,86	end of treatment	No	5.8	6,00	8,00
180,58	diagnosis	No	9,30	15,70	16,50
214,13	diagnosis	No	11.8	10.6	9.3
74,87	diagnosis	No	-	-	-
200,23	diagnosis	No	29,00	12.4	19.5
164,49	diagnosis	No	9.5	18.6	20,00
167,21	diagnosis and in the end of treatment	No	-	-	-
98,98	end of treatment	Yes	5.6	7.4	11.1
204,01	diagnosis	No	-	-	-
36,93	diagnosis	No	7.5	6,00	8.8
141,59	end of treatment	Yes	11,00	17,00	19,00
227,89	end of treatment	Yes	10,00	9.7	8.5
192,08	end of treatment	No	30.6	14,00	21.5
200,66	diagnosis	No	3.6	5.8	8,00
183,84	diagnosis	No	-	-	-
189,88	diagnosis	No	-	-	-
110,55	diagnosis	No	-	-	-
138,21	diagnosis	No	-	-	-
241,06	diagnosis	No	-	-	-
181,34	diagnosis	No	15.7	21.3	20.4
17,15	diagnosis	No	4.9	6.3	8.5
179,73	diagnosis	No	4,00	3.5	4.5
11,20	diagnosis	No	7.5	11.5	15,00
225,53	diagnosis	No	5,30		3,50
233,05	diagnosis	No	2.5		2,00

Age_(last_information_or_death)	Surgery	Biopsy	AP	TRV	CC
194,28	diagnosis	No	9.7	8.7	7.8
54,37	diagnosis	No	2,00		1.8
186,83	diagnosis	No	13.4	21,00	32,00
70,53	diagnosis	No	-	-	-
116,20	diagnosis	No	6.8	11,00	10.9
19,19	diagnosis	No	9.8	11.1	11.6
16,72	diagnosis	No	8.2	13.6	16,00
11,96	diagnosis	No	-	-	-
194,19	diagnosis and after three cycles of chemotherapy	No	1.3	.9	
216,33	diagnosis and in the end of treatment	No	-	-	-
141,20	end of treatment	Yes	-	-	-
217,15	diagnosis	No	8.7	7.4	6.5
